# Contact with primary care physicians among adults with pre-existing common mental health problems during the COVID-19 pandemic: a registry-based study from Norway

**DOI:** 10.1186/s12913-023-10108-3

**Published:** 2023-10-11

**Authors:** Pia Jensen, Christian Madsen, Lars Johan Hauge, Kristin Gustavson, Ingunn Olea Lund, Johanne Hagen Pettersen, Ann Kristin Skrindo Knudsen, Anne Reneflot, Ragnhild Eek Brandlistuen, Unnur Anna Valdimarsdóttir, Helga Ask, Ragnar Nesvåg

**Affiliations:** 1 Department of Mental Disorders, Norwegian Institute of Public Health, Oslo, Norway; 2 Department of Psychology, University of Oslo, Oslo, Norway; 3 Department of Disease Burden, Norwegian Institute of Public Health, Bergen, Norway; 4 Department of Mental Health and Suicide, Norwegian Institute of Public Health, Oslo, Norway; 5 Department of Child Health and Development, Norwegian Institute of Public Health, Oslo, Norway; 6 Centre of Public Health Sciences, Faculty of Medicine, School of Health Sciences, University of Iceland, Reykjavik, Iceland; 7 Unit of Integrative Epidemiology, Institute of Environmental Medicine, Karolinska Institutet, Stockholm, Sweden; 8 Department of Epidemiology, Harvard TH Chan School of Public Health, Boston, MA USA

**Keywords:** Covid-19, Mental health problems, Adults, Primary care, Health registry, Service utilization

## Abstract

**Background:**

During the COVID-19 pandemic, individuals with pre-existing mental health problems may have experienced additional stress, which could worsen symptoms or trigger relapse. Thus, this study aimed to investigate if the number of consultations with general practitioners (GPs) among individuals with a pre-existing common mental health problem during the pandemic differed from pre-pandemic years.

**Methods:**

Data on consultations with GPs among 18–65-year-olds registered with common mental health problems in 2017–2021 were retrieved from the Norwegian Control and Payment of Health Reimbursements Database. Based on data from the pre-pandemic years (2017–2019), we predicted the number of consultations per week for depression, anxiety disorder, phobia/obsessive–compulsive disorder (OCD), post-traumatic stress disorder (PTSD), and eating disorders during the pandemic (March 2020-December 2021) among individuals with pre-existing mental health problems. The forecasted and observed trends in GP consultations per week during the pandemic were stratified by diagnosis, gender, and age groups.

**Results:**

The observed number of consultations for anxiety disorder, PTSD, and eating disorders were significantly higher than forecasted during extended periods of the two pandemic years. The differences were largest for PTSD (on average 37% higher in men and 47% higher in women during the pandemic), and for eating disorders among women (on average 87% higher during the pandemic). There were only minor differences between the predicted and observed number of consultations for depression and phobia/OCD.

**Conclusions:**

During the pandemic, individuals with a recent history of mental health problems were more likely to seek help for anxiety disorder, PTSD, and eating disorders, as compared to pre-pandemic years.

**Supplementary Information:**

The online version contains supplementary material available at 10.1186/s12913-023-10108-3.

## Introduction

On March 11, 2020, the coronavirus disease 2019 (COVID-19) was declared a pandemic by the World Health Organization [1]. The measures to prevent the spread have not only had a considerable impact on physical and mental health outcomes [2], but also affected the delivery of mental health care [3]. During the first wave of the pandemic in Norway, many mental health facilities were forced to reduce or temporarily suspend their services [4, 5], which might have affected the access and utilization of such services.

Studies from the UK found a reduction in the number of primary care consultations for mental health problems during the first period of the pandemic [6, 7] and reduced incidences of primary care-recorded mental disorders [8, 9] among the general population. When looking at a longer period during the pandemic, some of the studies from the UK found that the number of primary care consultations for mental health problems remained lower than expected by the end of the study period compared to the pre-pandemic years [6–8]. In Norway, the number of general practitioner (GP) consultations for mental health problems increased during the spring and early summer of 2020 [10]. However, by July and August 2020, the level of consultations decreased towards pre-pandemic levels. By September 2020, the number of consultations accelerated, and the increase lasted throughout 2020.

The previous studies mentioned above have only investigated the first few months or the first year of the pandemic and focused on service utilization in the general population. During a public health crisis like the COVID-19 pandemic, it is also important to identify and investigate vulnerable groups who may be at greater risk of experiencing worse psychological outcomes [11]. Limited access to mental health services and the negative psychosocial effects of social distancing measures are likely to disproportionately affect individuals with a history of mental health problems [12, 13]. For instance, limited access to services or fear of seeking help could increase the risk of discontinuation or abrupt termination of mental health treatment, which can worsen mental health problems [14]. Lack of social support and activities that maintain mental health could further exacerbate psychiatric distress and affect overall functioning [15]. In addition, individuals with pre-existing mental health problems are also more susceptible to stress than the general population [16], which could increase relapse rates [17, 18]. A systematic review and meta-analysis based on data-collections done in 2020 did not find any evidence of an increase in symptoms of mental disorders among individuals with a pre-existing mental disorder [19]. However, it is unknown whether these findings can be generalized to the prolonged duration of the pandemic, when the strain on people with mental health problems may have been even greater. Consequently, it is important to investigate whether this group’s mental health care use changed during the pandemic.

To our knowledge, only a few studies have investigated the effect of the pandemic on service utilization among individuals with pre-existing mental health problems. Ridout et al. [20] used electronic health records in a large community, primarily an employer-based health system in the US, and investigated psychiatric service utilization in 2020 compared with 2019. They found that the number of patients with a pre-existing psychiatric diagnosis seeking help remained stable during the first year of the pandemic. In contrast, Janoczkin et al. [21] analyzed individuals presenting to one Emergency Department in America with psychiatric complaints between January 1-July 9 in 2019 and 2020 and found an increased prevalence of patients presenting with a history of previous psychiatric care. In addition to that these two studies have solely been based on data from 2020, another important limitation is their small and limited subject samples.

There is hence an important knowledge gap in the literature, as no published studies so far have used whole-population registry data to investigate the utilization of primary care for mental health problems during a prolonged period of the pandemic among adults with a recent history of mental health problems.

In Norway, all citizens registered as living in Norway in the National Population Register have the right to a General Practitioner (GP). GPs could be seen as gatekeepers to other health services, as they often are the patient’s first contact within the health care system, and the patient may contact their GP without a referral. Based on the patients’ needs, the GP can refer the patient to services in specialist health care, such as hospitals and outpatient clinics owned or financed by the government. In Norway, the treatment of moderate to severe mental disorders is given by specialist health care, and patients need a referral for help. In Norway, primary and specialist health care are linked treatment services, and increased access to specialist health care may lead to less demand for primary care and vice versa. During the pandemic, all GP offices remained open, although with strict restrictions regarding in-person consultations. At the start of the pandemic lockdown in March 2020, mental healthcare facilities were open for emergency care only, and patients were advised to wait or have electronic consultations (phone/video). However, the mental health care services adjusted their services to accommodate the pandemic situation and the level of restrictions.

The present study aims to estimate to what extent the number of primary care consultations for common mental health problems changed during the pandemic (2020 and 2021) compared to the pre-pandemic years (2018–2019) among individuals with a pre-existing mental health problem. To investigate changes in consultations before and during the pandemic, we created two different data sets (one for each time period), containing GP consultations for mental health problems among individuals with pre-existing mental health problems. We chose to focus on consultations for depressive disorder, anxiety disorder, phobia/obsessive–compulsive disorder (OCD), post-traumatic stress disorder (PTSD), and anorexia nervosa/bulimia (eating disorders), as these disorders have been hypothesized to be especially affected by the increased stress related to the COVID-19 pandemic [22–24]. The present study also adds to the existing literature by examining differences in service utilization patterns across disorders groups.

## Material and methods

### Data sources

We used data from the Norwegian emergency preparedness register for COVID-19 (Beredt C19) [25]. Beredt C19 was established at the Norwegian Institute of Public Health (NIPH) to rapidly obtain the necessary knowledge about the spread and consequences of the COVID-19 pandemic for public health in Norway. We used data from two electronic health registries included in Beredt C19: The Norwegian Control and Payment of Health Reimbursements Database (KUHR) and The National Population Register for 2017–2021.

#### The Norwegian Control and Payment of Health Reimbursements Database (KUHR)

To measure the number of GP consultations for mental health problems among individuals with a pre-existing mental health problem, we used data from KUHR, which provides information on bills from health services that have been reimbursed to doctors by the government. The following reimbursement codes were included in our definition of consultations: Consultation with GP during daytime or evening/night, consultation with out-of-hours primary care service during daytime or evening/night, e-consultation with GP during daytime or evening/night and e-consultation with out-of-hours primary care service during daytime or evening/night. For each consultation with a GP, the GP register one or more diagnostic codes that describes the patient’s clinical problem according to the International Classification of Primary Care system, 2nd edition (ICPC-2) [26]. Our data included all GP consultations among individuals aged 18–65 years during 2017–2021 that was coded with one of the following diagnostic codes: depressive disorder (P03; P76), anxiety disorder (P01; P74), phobia/OCD (P79), PTSD (P82), and anorexia nervosa/bulimia (P86, eating disorders).

#### The National Population Register

We used The National Population Register to include information about gender (male/female) and age group (18–24 years, 25–39 years, 40–65 years).

### Design

**Fig. 1:**
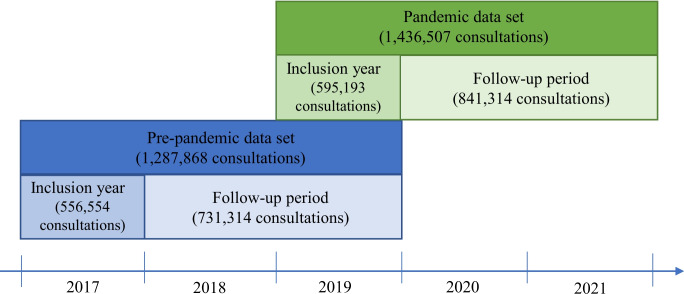
Illustration of the two data sets. Inclusion year refers to the year individuals had one or more consultations with a GP to be defined as having a pre-existing mental health problem in the two data sets

To compare the number of primary care consultations for mental health problems among individuals with common pre-existing mental health problems before and during the pandemic, we created a pre-pandemic data set and a pandemic data set. Each data set contained the total number of consultations per week during the period of interest for the pre-selected mental health codes retrieved from KUHR. In both data sets, only the consultations of individuals with pre-existing mental health problems were included. One individual could have multiple consultations during a week, and each consultation counts when estimating the total number of consultations per week. It is also possible that one individual's consultations are included in both data sets, as there is an overlap between the two data sets in 2019 (see Fig. 1).

#### Pre-pandemic data set

In the pre-pandemic data set (2017–2019), the criteria of having a pre-existing mental health problem were to have one or more consultations with a GP during 2017 that was coded with one of the following: Feeling depressed (P03), depressive disorder (P76), feeling anxious (P01), anxiety disorder (P74), phobia/OCD (P79), PTSD (P82) and/or eating disorders (P86). We included P03 and P01, as it is possible that these symptoms might develop into P76 and P74 later. In the analyses, we were only interested in GP consultations during 2017–2019 that were coded with depressive disorder (P76), anxiety disorder (P74), phobia/OCD (P79), PTSD (P82) and/or eating disorders (P86). We excluded P03 and P01 in the analyses, as these codes are characterized as symptoms in ICPC-2. In the analyses, we were only interested in conditions characterized as disorders, as these might affect an individual's daily life. All individuals who met the inclusion criteria in 2017 were followed in 2018–2019, and their number of GP consultations were included in the analyses.

#### Pandemic data set

In the pandemic data set (2019–2021), the criteria of having a pre-existing mental health problem were to have one or more consultations with a GP during 2019 that was coded with one of the same diagnostic codes as for the pre-pandemic data set. All individuals who met the inclusion criteria in 2019 were followed in 2020–2021, and their number of GP consultations were included in the analyses.

### Statistical analysis

All statistical analyses and data visualizations were conducted using R Studio version 1.4.1717 [27, 28]. The R package *boot* was used for simulations [29], and *ggplot2* was used to visualize the time series [30]. We conducted analyses stratified by gender and age group, and for each diagnostic code (P76, P74, P79, P82, P86) separately.

**Fig. 2:**
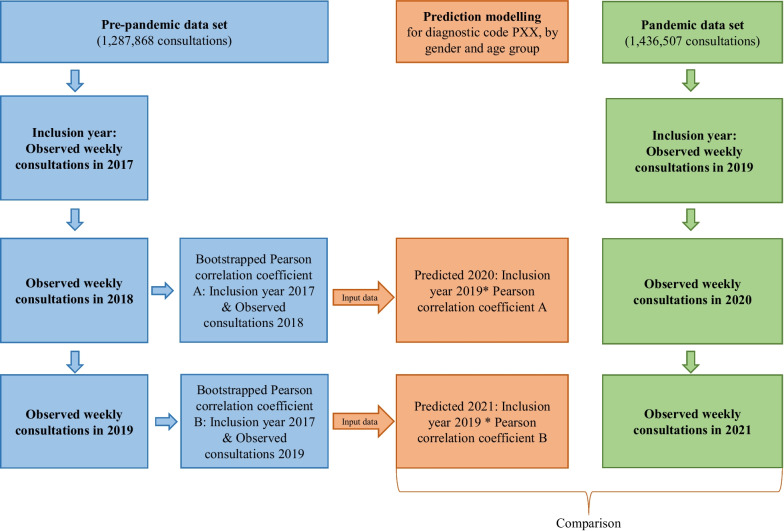
Illustration of the statistical analysis. Each analysis only includes the total number of consultations per week of one of the five ICPC-2 diagnoses of interest

First, we summarized the total number of consultations for each specific diagnostic code per week in the pre-pandemic data set (2017–2019) (see Fig. 2). Second, since we only had one observation period (2017–2019) to make predictions, we used bootstrap to estimate the mean Pearson correlation coefficient between the number of consultations per week in 2017 (inclusion year) and 2018 based on 10.000 randomly drawn samples from the data set with replacement. The same procedure, with estimating the mean Pearson correlation coefficient, was repeated for consultations in 2017 (inclusion year) and 2019. The mean Pearson correlation coefficients were treated as fixed values in the model. In the randomly drawn samples, the number of consultations per week varied, and the mean Pearson correlation coefficient was therefore estimated based on 10,000 different combinations of the data. Our model is based on the assumption that the variation we found in 2017–2019 is the same as the variation we could have found given more data from separate time periods before the pandemic. In other words, we assume that the variation in 2017–2019 represents the variation in any given three-year period, which we also could expect during 2019–2021. Hence, when drawing 10,000 random samples from the data, we simulated a sampling distribution of correlations between different years. This sampling distribution could then be used to draw inferences about what would happen in subsequent years if the pandemic did not affect the number of consultations.

To predict the number of consultations in 2020, the observed number of consultations per week during the inclusion year in the pandemic data set (2019) was multiplied with the mean Pearson correlation coefficient estimated between the years 2017 and 2018 from the pre-pandemic data set (see Fig. 2). The same procedure was repeated for predicting the number of weekly consultations in 2021, except for multiplying the observed number of consultations per week in 2019 (inclusion year in the pandemic data set) with the mean correlation coefficient estimated between the years 2017 and 2019 from the pre-pandemic data set. We multiplied the consultations during the inclusion year in the pandemic data set with the two correlation coefficients because 1) we assume that the associations between the number of consultations in different years should be constant across the two time periods if the pandemic had no effect, and 2) we expect the number of consultations to be maximum the same as in the inclusion year, or lower. This is based on the pre-pandemic data set, where we generally found a higher correlation between the inclusion year and the following year and a lower correlation between the inclusion year and the second year. By multiplying the correlation coefficients with the observed number of consultations during 2019, the predicted number of consultations in 2020 and 2021 was scaled to the number of consultations during the inclusion year in the pandemic data set.

Finally, we used Loess Regression with a 99.9% Confidence Interval (CI) on the predicted number of consultations per week in 2020 and 2021 to compare with the observed number of consultations per week in 2020 and 2021 from the pandemic data set. We chose to use 99.9% CI, as our analyses were based on only one observation period (the pre-pandemic data set) to make predictions. A smoothed line was applied to each time series plot (predicted and observed). The degree of smoothing was tested using the geom_smooth() function in the *ggplot2* package in R [30]. A span of 17% was found to be the optimal level for visualizing the time trends, which allowed both natural and seasonal irregularities in the time series while also showing an overall trend.

To calculate the mean difference between the observed and predicted number of consultations during the pandemic (2020–2021) for each diagnostic code, we first calculated the absolute difference between the observed and predicted number of consultation by using the abs() function. Lastly, to get the mean deviation from the predicted number of consultations, we divided the absolute difference with the total number of predicted consultations and multiplied it with 100. This percentage shows the mean difference between the observed and predicted number of consultations during the pandemic years. This procedure was repeated for each subgroup within each diagnostic code (gender and age groups).

In the beginning of January 2020, the Norwegian society was aware of an outbreak of a potentially lethal virus in China, and by the end of January 2020, there were several news articles about the serious consequences if the virus spread to Norway. Therefore, we regard 2020 as different from pre-pandemic years and decided not to include the pre-pandemic weeks in 2020 in our results. Since the aim of the study was to investigate changes in the number of mental health consultations in primary care among individuals with recent pre-existing mental health problems during the pandemic, we only show results from week 11 in 2020 and onward, as this was the week the Norwegian government implemented the first strict confinement measures.

### Ethics

This study was approved by The Regional Ethics Committee for Medical Research South-East Norway (June 25th, 2021, #267200).

## Results

### Study population

The pre-pandemic data set included 1,287,868 registered consultations for depression, anxiety, phobia/OCD, PTSD, and eating disorders during 2017–2019, within 176,514 unique patients (62% women) identified in the inclusion year. The pandemic data set included 1,436,507 consultations during 2019–2021 within 186,824 unique patients (62% women) identified in the inclusion year. For more information about number of consultations and demographic information on unique patients in the two data sets see Supplementary table S1 and S2 in Additional file 1.

### Anxiety disorder

**Fig. 3:**
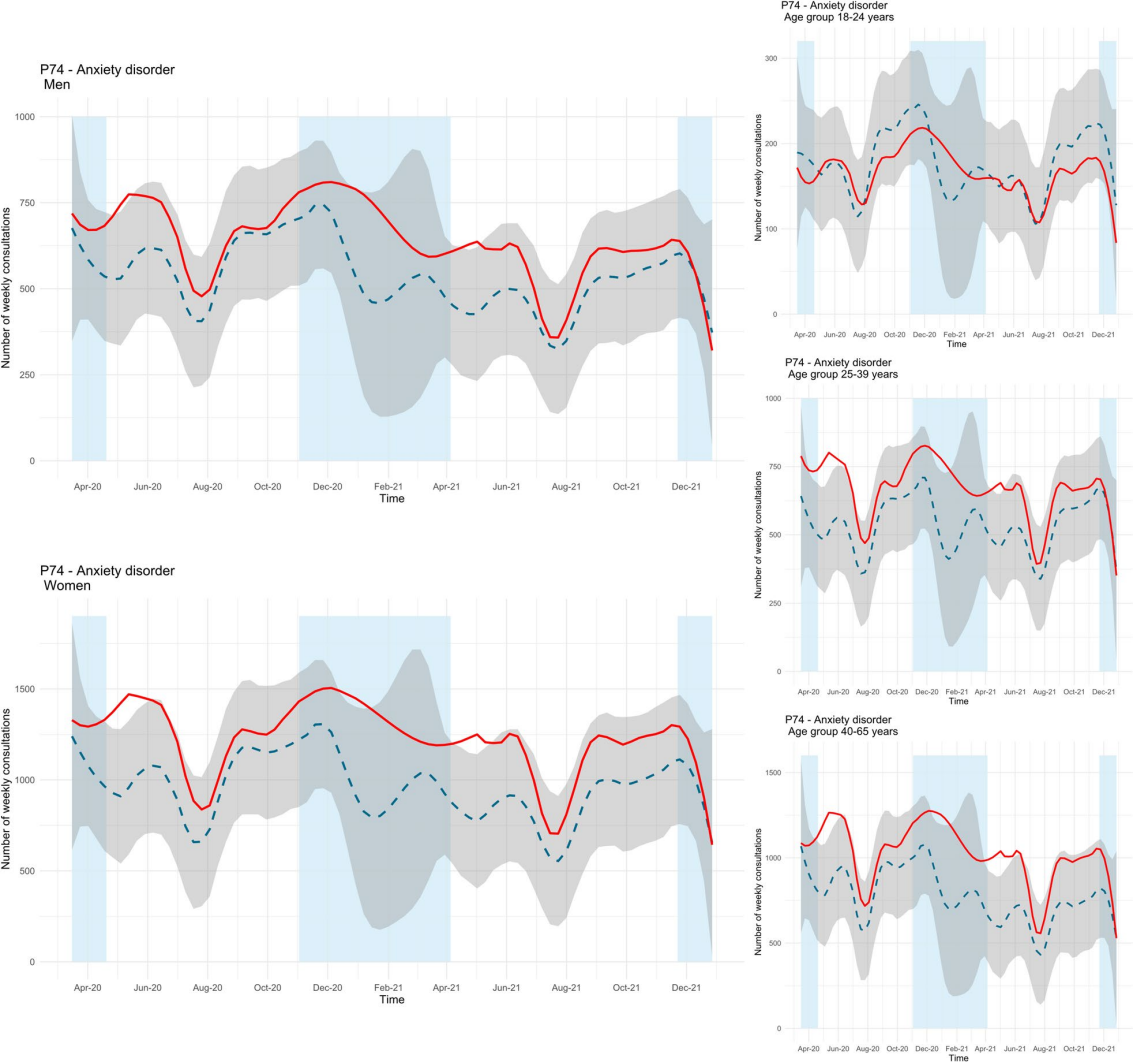
Time series plots for observed consultations (solid red line) for anxiety disorder (ICPC-2 code P74) across gender and age groups with forecast (dashed blue line, with 99.9% confidence interval in grey). Blue fields represent periods with strict social distancing measures from the Norwegian government [31]

The observed number of consultations for anxiety disorder among men was higher than predicted in May 2020 and May 2021 (Fig. 3, upper left panel). The mean difference between the observed and predicted trend during the study period was 19%. As with men, there was also a period with an increased number of consultations among women in May 2020 and May 2021, but also in January 2021 (Fig. 3, lower left panel). During the pandemic, the mean difference between the observed and predicted number of consultations was 27%. When divided by age groups, the pattern of more consultations during May 2020, January 2021 and May 2021 were evident in the age groups 25–39 years and 40–65 years, but not in 18–24-year-olds (Fig. 3, right panels). During the study period, the mean difference between the two trends were 13% in 18–24-year-olds, 25% in 25–39-year-olds, and 32% in 40–65-year-olds.

### PTSD

**Fig. 4:**
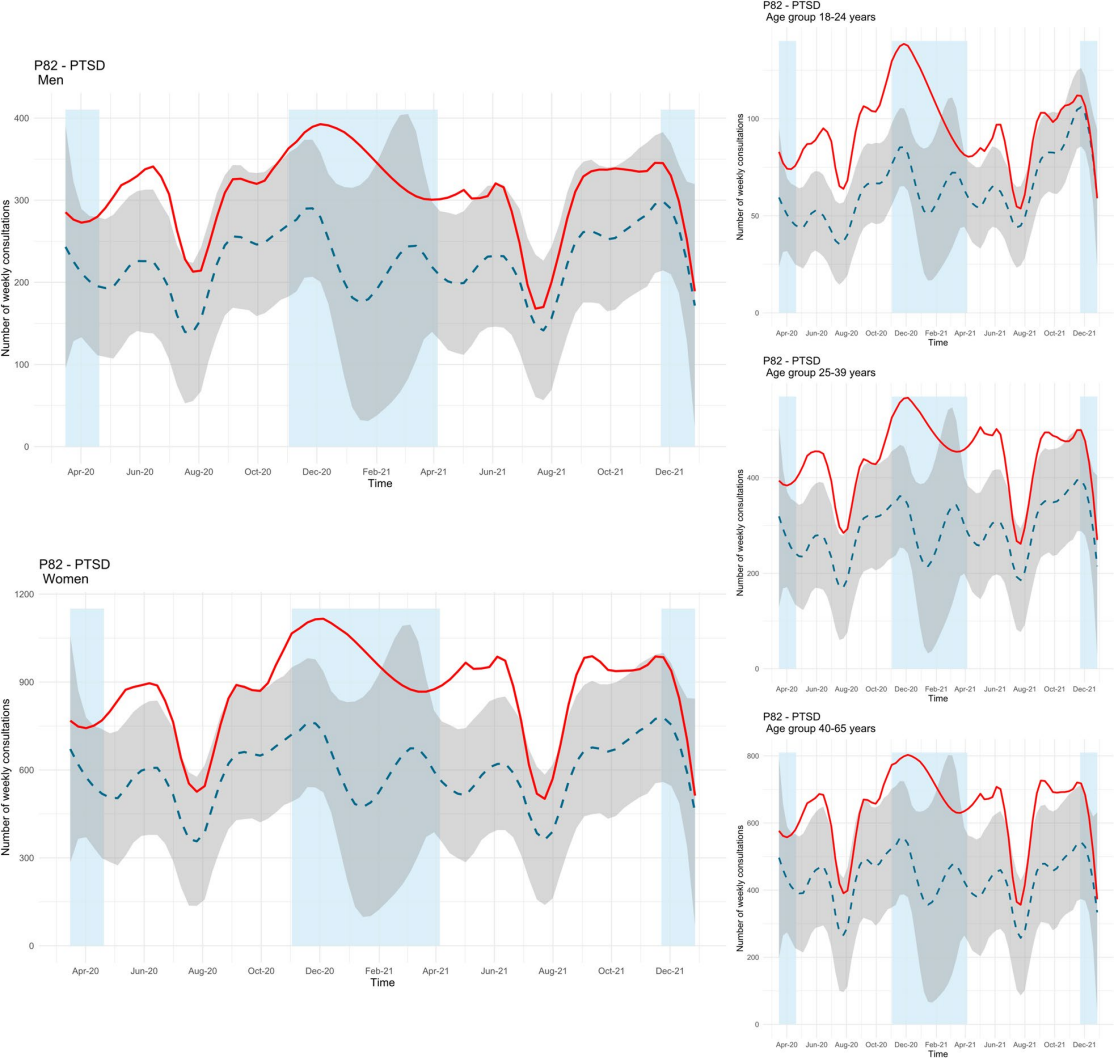
Time series plots for observed consultations (solid red line) for PTSD (ICPC-2 code P82) across gender and age groups with forecast (dashed blue line, with 99.9% confidence interval in grey). Blue fields represent periods with strict social distancing measures from the Norwegian government [31]

The observed number of consultations with a GP for PTSD was higher than predicted during extended periods of the pandemic across all groups. Among men, the periods with increased consultations were during spring/summer 2020, winter 2020, and spring 2021 (see Fig. 4, upper left panel). The mean difference between the observed and predicted trend was 37% during the study period. A similar pattern was also found for women, with increased consultations during spring/summer 2020, fall/winter 2020, and spring/summer 2021, but also during fall 2021 (see Fig. 4, lower left panel). Among women, the mean difference between the two trends was 47%. Similar periods with significant differences among women were also found across all age groups, but the youngest age group (18–24 years) had the longest period with more consultations than predicted (see Fig. 4, right panels). During the pandemic, the mean difference between the observed and predicted consultations was 49% among those aged 18–24 years, 53% among those aged 25–39 years, and 48% among those aged 40–65.

### Eating disorders

**Fig. 5:**
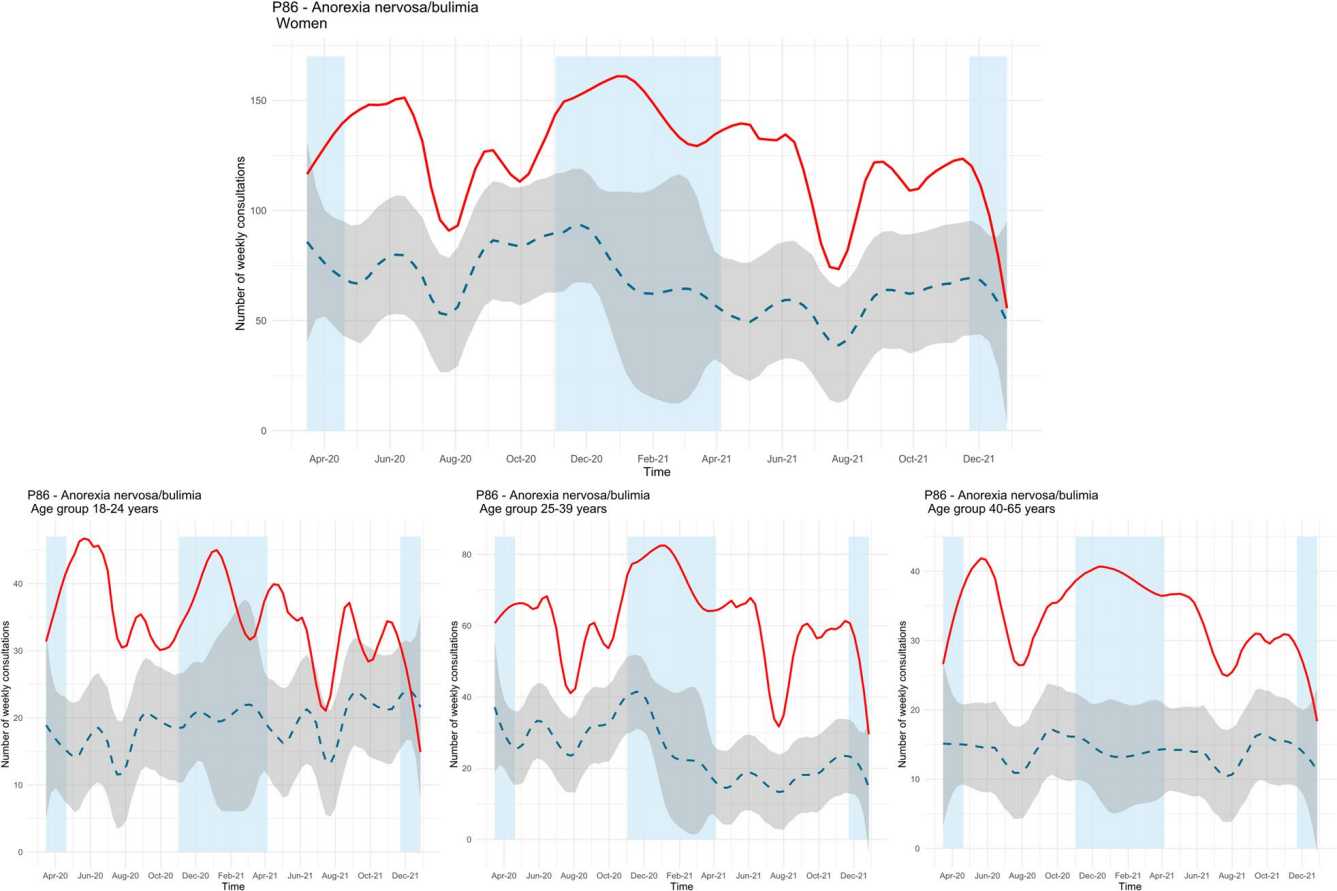
Time series plots for observed consultations (solid red line) for eating disorders (ICPC-2 code P86) among women and age groups with forecast (dashed blue line, with 99.9% confidence interval in grey). Blue fields represent periods with strict social distancing measures from the Norwegian government [31]

GP consultations for eating disorders (anorexia nervosa/bulimia) among men were low throughout the study period. Therefore, we only show results for women and different age groups. Except from the beginning of March 2020 and end of December 2021, the observed number of consultations with a GP for eating disorders among women were much higher than predicted (see Fig. 5, upper panel). Among women, the mean difference between the observed and predicted trend during the two pandemic years was 87%. The same increase in observed consultations were found across the two oldest age groups (25–39 years and 40–65 years). For the youngest age group, the number of consultations was significantly higher from the beginning of the restrictions (week 11, 2020) until February 2021. During the summer holiday and fall (mid-September to mid-October 2021) the levels decreased, and the difference was no longer significant. Throughout the study period, the mean difference in the number of observed and predicted consultations was 84% among the youngest age group, 148% among the middle age group, and 139% among the oldest age group (see Fig. 5, lower panels).

### Depression and phobia/OCD

During most of the study period, there were no significant differences in observed vs. predicted number of consultations for depressive disorder. However, there was a lower number of observed consultations in December 2021 among men and 25–39-year-olds, and significantly fewer consultations among 18–24-year-olds from September to December 2021 (Supplementary Figure S1 in Additional file 1). There were generally no significant differences between the observed and predicted number of consultations for phobia/OCD during the pandemic, except a significantly higher number of consultations during the spring of both 2020 and 2021 in the oldest age group (40–65 years) (Supplementary Figure S2 in Additional file 1).

## Discussion

Our study, which includes nationwide registry data from 2017–2021, indicates that the COVID-19 pandemic has differently affected service utilization for various mental health problems. Among individuals with pre-existing mental health problems, we found a massive increase in primary care consultations for PTSD and eating disorders during the pandemic compared to the pre-pandemic years. The increase in consultations for PTSD was most apparent among the youngest age group, while the increase in consultations for eating disorders was apparent among women and all age groups. We also found that the number of consultations for anxiety disorder were higher than predicted in time periods following strict social distancing measures. Further, we found no or minor changes in the number of consultations for depression and phobia/OCD.

Our results show that the number of GP consultations for mental health problems among individuals with a history of mental health problems increased or remained stable during the pandemic years. This indicates that primary care succeeded in providing and sustaining healthcare for this vulnerable group even during a national crisis. The general finding of increased use of primary care during the pandemic years among this group is in line with the study by Janoczkin et al. [21]. The increased use of primary care among some individuals with a pre-existing mental health problem might be due to several factors. Firstly, the experience of symptom worsening and risk of relapse in this group might increase the service utilization, which have been reported in several reviews [32–34]. However, one review did not find any symptom worsening among individuals with pre-existing mental health problems [19]. Secondly, the increased number of GP consultations among our sample could be the result of displacing nurses from low threshold services for mental health care in municipalities for corona-related tasks, as this might have reduced municipal mental health care provision. Thirdly, it could be the result of reduced access to specialist health care [4], which could worsen mental health problems and increase the need for help in primary care. Lastly, the increased number of GP consultations for mental health problems might reflect that GP offices have been open during the pandemic, with increased use of e-consultations. E-consultations likely made it easier for GPs to follow their patients more regularly, which could lead to more consultations for mental health problems [35]. The new daily routines and work-situation caused by the strict confinement measures could also influence the use of GP consultations, due to more time at home, less meetings, and more flexible work-schedules. However, the present study cannot investigate any causal relationships, and it is likely that multiple factors operate at the same time.

We found a higher number of observed GP consultations than predicted for anxiety disorder after the first and second periods with strict contact-reducing measures (except in the youngest age group). In addition, we also found increased consultations during the second period with strict contact-reducing measures among women and the two oldest age groups. Our result contradicts studies that have found a reduction in the number of primary care consultations for anxiety [6, 7]. The different result might be due to the fact that Lemanska et al. [7] and Mansfield et al. [6] investigated primary care consultations for anxiety in the general population and not specifically among individuals with a history of mental health problems. The finding of increased consultations with GPs after periods with strict restrictions might indicate that some people were reluctant to use mental health services during the strict periods and that the increase was due to a spill-over effect after the strict measures were alleviated. One explanation could be that individuals were afraid of getting infected with the virus at the GP office or stayed at home due to public health restrictions [4, 32]. Since we also found increased number of consultations during the second strict period among women and the two oldest age groups, this might indicate that social distancing measures triggered or exacerbated symptoms among some, but not all, individuals, which have been previously documented [36, 37].

Further, we found a large increase in the number of consultations for PTSD in several periods during both pandemic years. The increase was observed after the first strict period, and during and after the second strict period among both genders. In addition, there were also a period with increased number of consultations during the fall of 2021 among women and all age groups. Our results specifically indicate that the youngest age group (18–24 years) have been more affected by the pandemic, compared to the older age groups. The youngest age group had the largest and longest period where the consultations exceeded the predicted levels, which lasted from April 2020-February 2021. This is in line with a Norwegian study on the general population that found that the risk of fulfilling the symptom criteria for PTSD was associated with lower age [38]. The finding of increased consultations for PTSD adds to the literature using survey data, as some studies have found that a considerable proportion of psychiatric patients have reported severe PTSD-like symptoms during the initial stage of the pandemic [39, 40]. One possible explanation for the present finding could be that individuals with pre-existing mental health problems have a higher risk of developing PTSD than individuals without a history of mental health conditions, as were documented in a Norwegian sample [41]. Therefore, the pandemic may have increased stress levels among individuals with a pre-existing mental health problem, which in turn might have triggered or exacerbated symptoms of PTSD. However, there are debates as to whether the COVID-19 pandemic can be considered a traumatic event and fulfill DSM-5's Criterion A for receiving a PTSD diagnosis [42]. It is therefore more likely that the pandemic increased symptoms among some individuals who already struggled with PTSD before the pandemic. Another explanation could be a biological effect through the COVID-19 virus itself or via inflammatory or other immune processes [43], as increased plasma concentration of the inflammatory marker C-reactive protein (CRP) are associated with PTSD symptoms [44]. However, estimates from the Norwegian Institute of Public Health suggest that less than 10% of the Norwegian population had been infected by the coronavirus by the end of 2021 [45], compared to 40–70% of the population by the end of June 2022 [46].

Lastly, we found a massive increase in the number of observed consultations for eating disorders among women and all age groups. Except from the beginning of the pandemic in 2020, the end of 2021, and between February 2021-March 2021 among the youngest age group (18–24 years), the observed number of consultations exceeded the predicted number of consultations during both pandemic years. This is the opposite of what Mansfield et al. [6] found in their study on primary care consultations in the general population, but supports findings from specialized health care on service use [47–49]. The divergent finding of Mansfield et al. [6] might be due to sample differences, as our finding is in line with studies from specialized health care, where individuals have an existing mental disorder [47–49]. A systematic review reported symptom worsening among individuals with eating disorders during the first year of the pandemic [33], but the variation in reported symptom worsening is large [50]. When looking at the literature on children and adolescents, a Norwegian registry study found a major increase in the use of mental health services (both primary- and specialist care) for eating disorders during the first year of the pandemic [51]. Our study extends the research findings on children and adolescents and shows that the increased use of health care for eating disorders also is evident among adults with a history of mental health problems. Symptoms worsening and the increased number of GP consultations for eating disorders during the pandemic could be the result of isolation, loss of structure, changes to routines, and negative influence of social media [52].

In all time series plots, there was a sharp decline in the number of observed consultations during December 2021, while this was not the case for December 2020. This is a statistical artefact probably due to some consultations in December 2021 being registered in January 2022, and thus not included in the pandemic data set.

There are several strengths of this study. Firstly, the use of data from KUHR captures all patient encounters with publicly funded GPs in Norway, due to population coverage and specifically assigned GPs for all Norwegian citizens. Secondly, we had access to data from both 2020 and 2021, as a major limitation of the existing literature on the effects of the pandemic on mental health is the use of data from only 2020. Thirdly, our study design is based on an identical statistical procedure for the two data sets. Individuals with a pre-existing mental health problem were identified before the pandemic in both data sets (2017 and 2019). In addition, the number of consultations among these individuals was examined over two years, either before the pandemic (2018–2019) or during the pandemic (2020–2021). Fourthly, by measuring the use of primary care, we may have captured more individuals who have experienced mental health problems in our study population, as there is no need for a referral to get in contact with a GP or a risk of being rejected. This might give a better indication of how the pandemic has affected mental health compared to using data from specialist health care, which has capacity problems and is based on referrals.

Our study also has some limitations. Firstly, we only had access to data from 2017–2021. Thus, we only had one observation period before the outbreak of the COVID-19 virus. However, by using bootstrap we tried to compensate for the lack of access to more time periods before the pandemic when estimating the correlation coefficients used to make predictions. Secondly, we defined individuals as having a pre-existing mental health problem in the two data sets based on one inclusion year (2017 and 2019 respectively). Hence, we do not know if the patients defined as having a pre-existing mental health problem in our two data sets had frequent contact with GPs prior to the inclusion years. However, by relying on one inclusion year, we made the definition of pre-existing mental health problems identical in the two data sets. Thirdly, we assume that the pre-pandemic data set reflects a normal service use pattern and that the major difference between the two data sets is associated with the pandemic. In other words, we do not consider whether the mental health problems' prevalence or service use may have changed due to other factors during the pandemic. Fourthly, our prediction model is based on the assumption that the number of weekly GP consultations during the pandemic years are maximum the same or less than the consultations during the pre-pandemic years. In other words, we assume a stable development of GP consultations over time. However, it is likely that the trend of consultations is more nuanced and varies between years. Despite this, the predicted number of consultations for depression and phobia/OCD largely overlapped with the observed number of consultations, which indicates that our prediction model was able to make good predictions. Lastly, we rely on mental health problems diagnosed by GPs in routine care settings [53]. However, one study found that diagnoses in health registries (including KUHR) have moderate sensitivity and excellent specificity [54]. Moreover, we have no reason to believe that the diagnostic practice among GPs for mental health problems have changed during the pandemic.

## Conclusions

This study demonstrates that the COVID-19 pandemic has affected service utilization for mental health problems in various ways. Among some individuals with a pre-existing mental health problem, the use of primary care increased during the two pandemic years. Specifically, individuals with anxiety, PTSD, and eating disorders have been most affected by the pandemic and may have experienced clinical deterioration and relapse. In comparison, the pattern of service use among individuals with depression or phobia/OCD generally remained unchanged during the pandemic. Our study has implications for mental health care during a future crisis, as we have identified groups of mental health problems that might need special attention during a global stressor such as a pandemic.

### Supplementary Information

**Additional file 1: Supplementary table S1.** Detailed information about total number of consultations for mental health problems with general practitioners before and during the COVID-19 pandemic.** Supplementary table S2. **Demographic information about individuals registered with mental health problems at their general practitioner before and during the COVID-19 pandemic.** Supplementary figure S1. **Time series plots for observed consultations (solid red line) for depression (ICPC-2 code P76) across gender and age groups with forecast (dashed blue line, with 99.9% confidence interval in grey). Blue fields represent periods with strict social distancing measures from the Norwegian government [1].** Supplementary figure S2.** Time series plots for observed consultations (solid red line) for phobia/OCD (ICPC-2 code P79) across gender and age groups with forecast (dashed blue line, with 99.9% confidence interval in grey). Blue fields represent periods with strict social distancing measures from the Norwegian government [1].

## Data Availability

The data used in this study contain individual-level linked information from public health registries as part of Beredt C19. Data are not available for external use due to legal restrictions, but researchers may request linked data from the same health registries used in this study by filling out an electronic application at Helsedata.no (https://helsedata.no/en/).

## Abbreviations

COVID-19: Coronavirus disease 2019
GP: General Practitioner
OCD: Obsessive-compulsive disorder
PTSD: Post-traumatic stress disorder
Beredt C19: Norwegian emergency preparedness register for COVID-19
KUHR: The Norwegian Control and Payment of Health Reimbursements Database
ICPC-2: International Classification of Primary Care system, 2^nd^ edition
