# Supplementary material for: Contact with primary care physicians among adults with pre-existing common mental health problems during the COVID-19 pandemic: a registry-based study from Norway
Source: BMC Health Serv Res. 2023 Oct 11;23:1085. doi: 10.1186/s12913-023-10108-3 (PMC10568894; doi:10.1186/s12913-023-10108-3)
Supplement: Supplementary file 1 — Additional file 1: Supplementary table S1. Detailed information about total number of consultations for mental health problems with general practitioners before and during the COVID-19 pandemic. Supplementary table S2. Demographic information about individuals registered with mental health problems at their general practitioner before and during the COVID-19 pandemic. Supplementary figure S1. Time series plots for observed consultations (solid red line) for depression (ICPC-2 code P76) across gender and age groups with forecast (dashed blue line, with 99.9% confidence interval in grey). Blue fields represent periods with strict social distancing measures from the Norwegian government [1]. Supplementary figure S2. Time series plots for observed consultations (solid red line) for phobia/OCD (ICPC-2 code P79) across gender and age groups with forecast (dashed blue line, with 99.9% confidence interval in grey). Blue fields represent periods with strict social distancing measures from the Norwegian government [1]. [file 12913_2023_10108_MOESM1_ESM.docx]

Supplementary Information

# 1. Information about total number of consultations in data sets

**Supplementary table S1.** Detailed information about total number of consultations for mental health problems with general practitioners before and during the COVID-19 pandemic.

| Data set | Variable |  | N | % |
| --- | --- | --- | --- | --- |
| Pre-pandemic | Consultations during 2017-2019 |  | 1,287,868 | 100 |
|  |  |  |  |  |
|  | Consultations during inclusion year 2017 |  | 556,554 | 43 |
|  |  |  |  |  |
|  | Gender | Men | 457,111 | 35 |
|  |  | Women | 830,757 | 65 |
|  |  |  |  |  |
|  | ICPC-2-codes | P74 Anxiety disorder | 272,470 | 21 |
|  |  | P76 Depressive disorder | 833,280 | 65 |
|  |  | P79 Phobia/OCD | 75,900 | 6 |
|  |  | P82 PTSD | 137,456 | 11 |
|  |  | P86 Eating disorder | 18,008 | 1 |
|  |  |  |  |  |
|  | Number of included P-codes | 1 P-code | 1,240,321 | 96 |
|  |  | 2 P-codes | 45,881 | 4 |
|  |  | 3 P-codes | 1,633 | < 1 |
|  |  | >3 P-codes | 33 | < 1 |
|  |  |  |  |  |
| Pandemic | Consultations during 2019-2021 |  | 1,436,507 | 100 |
|  |  |  |  |  |
|  | Consultations during inclusion year 2019 |  | 595,193 | 41 |
|  |  |  |  |  |
|  | Gender | Men | 500,405 | 35 |
|  |  | Women | 936,102 | 65 |
|  |  |  |  |  |
|  | ICPC-2-codes | P74 Anxiety disorder | 324,324 | 23 |
|  |  | P76 Depressive disorder | 896,333 | 62 |
|  |  | P79 Phobia/OCD | 83,701 | 6 |
|  |  | P82 PTSD | 183,409 | 13 |
|  |  | P86 Eating disorder | 20,414 | 1 |
|  |  |  |  |  |
|  | Number of included P-codes | 1 P-code | 1,367,803 | 95 |
|  |  | 2 P-codes | 65,786 | 5 |
|  |  | 3 P-codes | 2,868 | < 1 |
|  |  | >3 P-codes | 50 | < 1 |
| Abbreviations: ICPC-2, International Classification of Primary Care system, 2^nd^ Edition  P-codes, Psychological codes | | | | |

# 2. Information about unique individuals in data sets

**Supplementary table S2.** Demographic information about individuals registered with mental health problems at their general practitioner before and during the COVID-19 pandemic.

|  |  | Pre-pandemic  (Inclusion year 2017) | | Pandemic  (Inclusion year 2019) |  |
| --- | --- | --- | --- | --- | --- |
| Variable |  | N | % | N | % |
| Total number of unique individuals |  | 176,514 | 100 | 186,824 | 100 |
|  |  |  |  |  |  |
| Gender | Men | 66,775 | 38 | 70,661 | 38 |
|  | Women | 109,739 | 62 | 116,163 | 62 |
|  |  |  |  |  |  |
| Age groups | 18-24 years | 24,380 | 14 | 26,636 | 14 |
|  | 25-39 years | 62,052 | 35 | 68,848 | 37 |
|  | 40-65 years | 90,082 | 51 | 91,340 | 49 |
|  |  |  |  |  |  |
| Relationship status* | Unmarried | 88,387 | 50 | 101,078 | 54 |
|  | Married | 50,786 | 29 | 50,437 | 27 |
|  | Separated/Divorced | 33,835 | 19 | 32,290 | 17 |
|  | Widow/Widower | 3,296 | 2 | 2,803 | 2 |
|  |  |  |  |  |  |
| Educational attainment** | Grade school | 53,273 | 30 | 57,228 | 31 |
|  | High school | 63,647 | 36 | 65,068 | 35 |
|  | University/College | 54,884 | 31 | 59,675 | 32 |
|  | No completed education | 380 | < 1 | 369 | < 1 |
|  |  |  |  |  |  |
| Student enrolled in higher education | Currently a university/college student | 12,410 | 7 | 14,012 | 8 |

*Missing: Pre-pandemic = 210 (< 1%), Pandemic = 216 (< 1%)

**Missing: Pre-pandemic = 4,330 (2%), Pandemic = 4,484 (2%)

# 3. Depressive disorder (P76) across gender and age groups


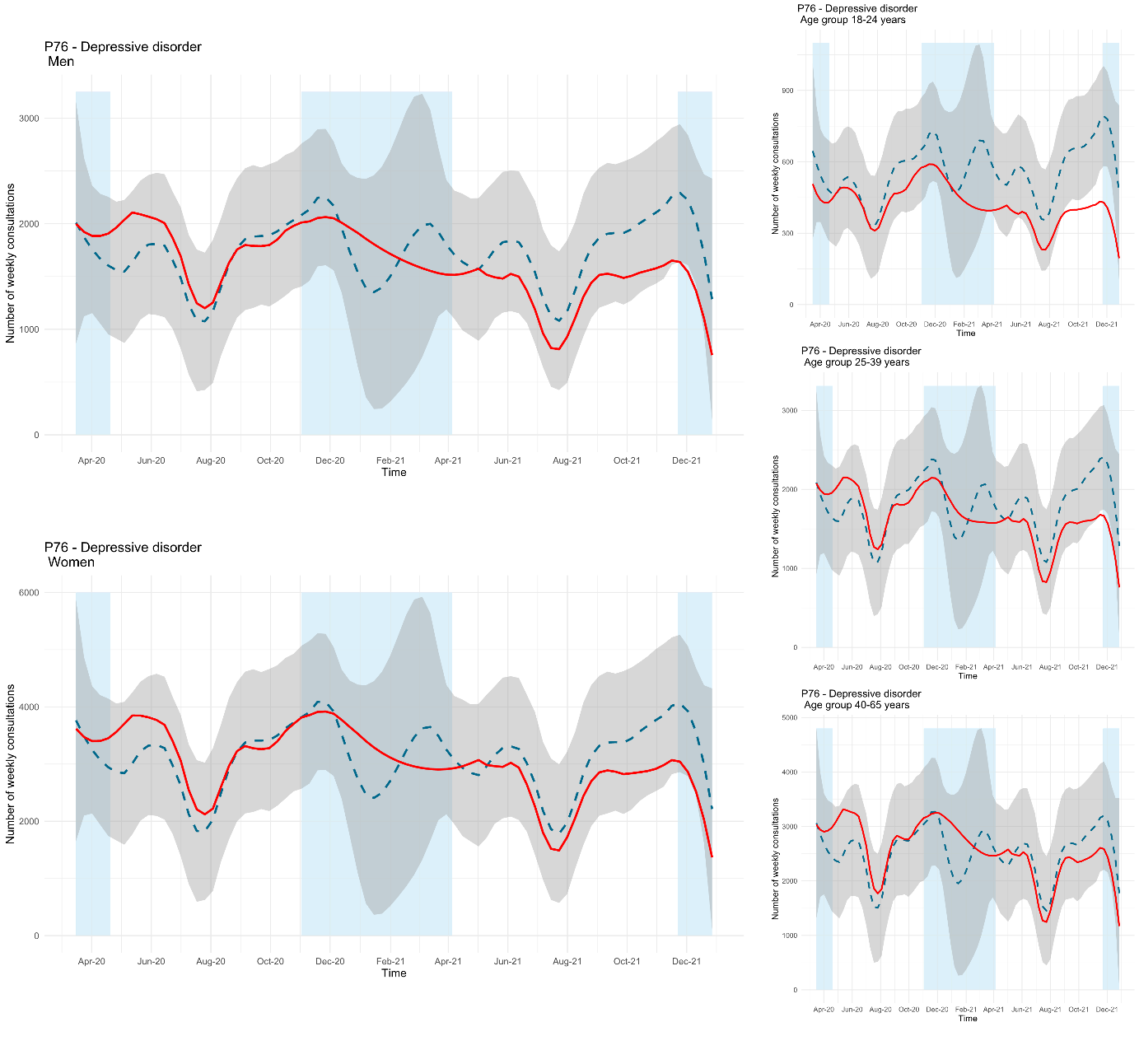


**Supplementary figure S1.** Time series plots for observed consultations (solid red line) for depression (ICPC-2 code P76) across gender and age groups with forecast (dashed blue line, with 99.9% confidence interval in grey). Blue fields represent periods with strict social distancing measures from the Norwegian government (1)

# 4. Phobia/OCD (P79) across gender and age groups

**
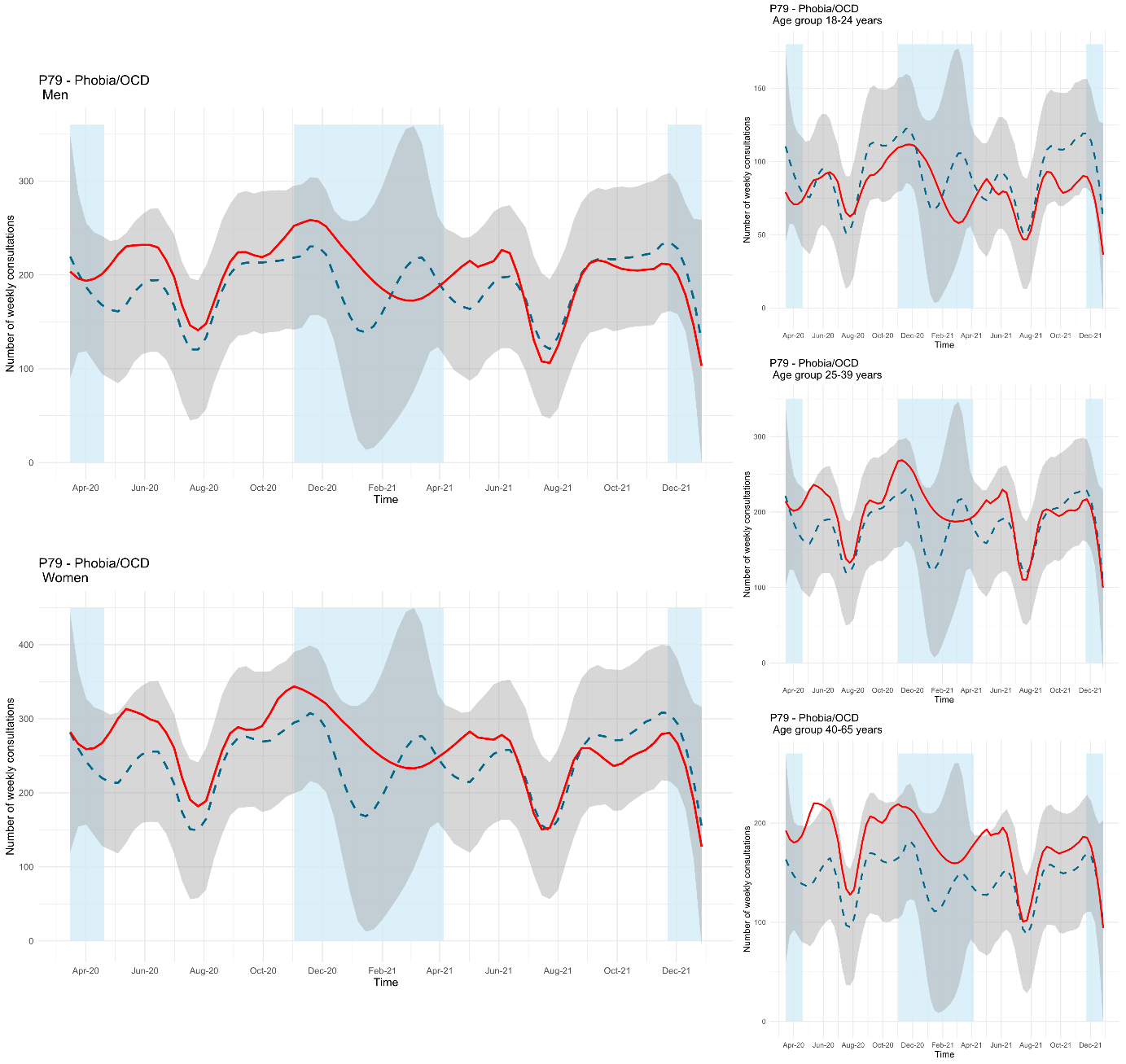
**

**Supplementary figure S2.** Time series plots for observed consultations (solid red line) for phobia/OCD (ICPC-2 code P79) across gender and age groups with forecast (dashed blue line, with 99.9% confidence interval in grey). Blue fields represent periods with strict social distancing measures from the Norwegian government (1)

**References**

1. Regjeringen. Tidslinje: myndighetenes håndtering av koronasituasjonen: Regjeringen; 2020 [Available from: <https://www.regjeringen.no/no/tema/Koronasituasjonen/tidslinje-koronaviruset/id2692402/>.
